# Developmental dyslexia susceptibility genes *DNAAF4*, *DCDC2*, and *NRSN1* are associated with brain function in fluently reading adolescents and young adults

**DOI:** 10.1093/cercor/bhae144

**Published:** 2024-04-12

**Authors:** Nea Rinne, Patrik Wikman, Elisa Sahari, Juha Salmi, Elisabet Einarsdóttir, Juha Kere, Kimmo Alho

**Affiliations:** Department of Psychology and Logopedics, University of Helsinki, Haartmaninkatu 3, 00014 Helsinki, Finland; Department of Psychology and Logopedics, University of Helsinki, Haartmaninkatu 3, 00014 Helsinki, Finland; Department of Psychology and Speech-Language Pathology, University of Turku, Assistentinkatu 7, 20500 Turku, Finland; Department of Neuroscience and Biomedical Engineering, Otakaari 3, Aalto University, (AALTO), P.O. BOX 00076, Espoo, Finland; Science for Life Laboratory, Department of Gene Technology, KTH-Royal Institute of Technology, SE-171 21, Solna, Sweden; Department of Biosciences and Nutrition, Karolinska Institutet, H7 Medicin, Huddinge, Sweden; Folkhälsan Research Center, and Stem Cells and Metabolism Research Program (STEMM), University of Helsinki, PL 63, Haartmaninkatu 8, Helsinki, Finland; Department of Psychology and Logopedics, University of Helsinki, Haartmaninkatu 3, 00014 Helsinki, Finland; Advanced Magnetic Imaging Centre, Aalto NeuroImaging, Aalto University, Espoo, Finland

**Keywords:** developmental dyslexia, general population, imaging genetics, neuroimaging, single nucleotide variation

## Abstract

Reading skills and developmental dyslexia, characterized by difficulties in developing reading skills, have been associated with brain anomalies within the language network. Genetic factors contribute to developmental dyslexia risk, but the mechanisms by which these genes influence reading skills remain unclear. In this preregistered study (https://osf.io/7sehx), we explored if developmental dyslexia susceptibility genes *DNAAF4*, *DCDC2*, *NRSN1*, and *KIAA0319* are associated with brain function in fluently reading adolescents and young adults. Functional MRI and task performance data were collected during tasks involving written and spoken sentence processing, and DNA sequence variants of developmental dyslexia susceptibility genes previously associated with brain structure anomalies were genotyped. The results revealed that variation in *DNAAF4*, *DCDC2*, and *NRSN1* is associated with brain activity in key language regions: the left inferior frontal gyrus, middle temporal gyrus, and intraparietal sulcus. Furthermore, *NRSN1* was associated with task performance, but *KIAA0319* did not yield any significant associations. Our findings suggest that individuals with a genetic predisposition to developmental dyslexia may partly employ compensatory neural and behavioral mechanisms to maintain typical task performance. Our study highlights the relevance of these developmental dyslexia susceptibility genes in language-related brain function, even in individuals without developmental dyslexia, providing valuable insights into the genetic factors influencing language processing.

## Introduction

Reading skills rely on specialized subnetworks in the brain, primarily within the left hemisphere language network (see e.g. Friederici 2011). Full maturity of reading skills is reached during adolescence and young adulthood, and the development is linked to concordant gray matter changes and increased white matter density (Kristanto et al. 2020). Approximately 5–10% of children struggle to attain adequate reading skills (Lyytinen 2008), which are essential in our daily lives. Developmental dyslexia (DD), the most common learning disability (Lerner 1989), is characterized by difficulties in acquiring proficient reading skills with a particular impact on the phonetic aspects of reading (Peterson and Pennington 2015).

Neuroimaging studies have identified brain anomalies related to reading skills in different subnetworks within the language network (Martin et al. 2016; Ramus et al. 2018). More specifically, DD is associated with functional and structural anomalies in the major nodes of the language network, encompassing the left inferior frontal gyrus (IFG), middle temporal gyrus (MTG), and intraparietal lobule, as well as anomalies in the structural connections between these areas (Schulz et al. 2008; Vandermosten et al. 2012; Martin et al. 2016). Postmortem studies, in turn, have found histological anomalies, such as ectopias, predominantly in the left hemisphere perisylvian regions, in people with DD (Galaburda et al. 1985). The structural and functional anomalies in temporal and parietal regions seem to persist regardless of age and language (Vandermosten et al. 2016; Xia et al. 2017), indicating an etiological and primary involvement in DD. In contrast, the anomalies in prefrontal brain areas are suggested to be related to reading performance and not the etiology of DD per se (Vandermosten et al. 2016).

In the field of imaging genetics of DD, various genes have been associated with different endophenotypes of the brain reading network, with *DCDC2* and *KIAA0319* emerging as the most widely studied genes in this context (reviewed by Erbeli et al. 2022 and Thomas et al. 2021). In the present study, we focus on the DD susceptibility genes *DNAAF4*, *DCDC2*, *KIAA0319*, and *NRSN1* since there is emerging evidence that variation in *DNAAF4*, *DCDC2* and *KIAA0319* is associated with white matter volume in language-related neural pathways connecting the left MTG and intraparietal sulcus (IPS) (Darki et al. 2012; Eicher et al. 2016). *NRSN1* was chosen based on a study by Skeide et al. (2016) associating allelic variation in this gene with gray matter volume in the visual word form area.

The single nucleotide variations (SNVs) studied here have not been reported in genome-wide association studies (GWASs) on dyslexia (reviewed by Erbeli et al. 2022), although genes with similar functions related to axon guidance pathway did reach corrected replication level significance in GWAS (Doust et al. 2022). However, DD-related GWASs face challenges in identifying SNVs with genome-wide significance. Due to necessary massive multiple comparison corrections, GWASs may be underpowered in detecting genetic variants with subtle effects such as the ones reported here (Wilkening et al. 2009). Furthermore, issues in standardizing DD identification across databases have hindered progress. It is also possible that the effects identified in candidate gene studies are specific to certain populations. Our primary aim was to study brain function associated with genes involved in neuronal migration that have been previously linked to DD, as well as reading performance and structural brain differences even in proficient readers. This approach allowed us to shed light on the mechanisms explaining interindividual differences in reading abilities.

These DD susceptibility genes appear to account for structural alterations in the left hemisphere temporal and parietal regions in neurotypical individuals (reviewed by Landi and Perdue 2019). For example, Darki et al. (2012, 2014) found that three SNVs, namely, rs3743204 (*DNAAF4*, previously called *DYX1C1*), rs793842 (*DCDC2*), and rs6935076 (*KIAA0319*), were associated with white matter volume in the left MTG and IPS in non-dyslexic 6–41-year-olds. Skeide et al. (2016), in turn, found an association between *NRSN1* and gray matter volume in the visual word form area (VWFA) of the left occipitotemporal cortex in 5–12-year-old preliterate and literate children. Apart from structural studies, Cope et al. (2012) reported a link between variation in *DCDC2* and brain function during reading. In addition, a cluster of three genes—containing *KIAA0319*—has been associated with functional asymmetry of the superior temporal sulcus (Pinel et al. 2012).

The DD susceptibility genes targeted here appear to be associated with essential neurodevelopmental processes such as neuronal migration (reviewed by Thomas et al. 2021). Specifically, *DNAAF4*, *DCDC*2, and *KIAA031*9 are ciliary genes with a role in neural migration (Meng et al. 2005; Massinen et al. 2011; Tammimies et al. 2012; Diaz et al. 2022; Bieder et al. 2023). *DNAAF4* seems to also play a role in estrogen signaling (Massinen et al. 2009) and *KIAA0319* and *NRSN1* in neurite growth (Nakanishi et al. 2006; Franquinho et al. 2017). These processes may have far-reaching implications for literacy skills, spanning both typical and atypical ranges. This is further supported by variation in *DCDC2* and *KIAA0319* being associated with reading skills in the general population (Paracchini et al. 2008; Lind et al. 2010). Given the link between these DD susceptibility genes and structural changes in brain regions important to reading and DD (Darki et al. 2012, 2014; Skeide et al. 2016), we posited that variation in these genes might also contribute to interindividual functional differences in these brain regions.

Our aim was to study how the DD susceptibility genes *DNAAF4* (previously named *DYX1C1*), *DCDC2*, *NRSN1*, and *KIAA0319* are associated with brain function in fluently reading adolescents and young adults (*n* = 179, age 13–25 years). We approached this by combining genetic data with functional magnetic resonance imaging (fMRI) and performance data from tasks requiring semantic processing of written and spoken sentences. We chose this set of genes, as these have been associated with brain structure and reading performance, not only in DD, but also in neurotypical individuals (see Table 1). However, a gap exists in understanding their impact on brain function. The SNVs rs3743204 (in *DNAAF4*), rs793842 (in *DCDC2*), and rs6935076 (in *KIAA0319*) were chosen based on Darki et al. (2012, 2014), rs9461045 (in *KIAA319*) based on Eicher et al. (2016), and rs10946672 (in *NRSN1*) based on Braineac database (http://www.braineac.org/).

**Table 1:** Summary of genetic associations with imaging phenotypes.

Gene	Function	Imaging phenotype	Brain region	Population	Reference
*DNAAF4 (DYX1C1)*	Ciliary function, neural migration, estrogen signaling	White matter volume	Left temporoparietal region; white matter tracts connecting the **left middle temporal gyrus** and **intraparietal lobule**	Typically reading children and young adults	Darki et al. 2012, 2014
		Brain function—EEG	Frontal regions	Typically developing children	Müller et al. 2017
*DCDC2*	Ciliary function, neural migration, microtubule regulation	White matter volume	Left temporoparietal region; white matter tracts connecting the **left middle temporal gyrus** and **intraparietal lobule**	Typically reading children and young adults	Darki et al. 2012, 2014
		Gray matter volume	Reading-related brain regions including the **left middle temporal gyrus**, **inferior parietal lobule**, and **inferior frontal gyrus**	Typically reading adults	Meda et al. 2008
		Brain function—fMRI	Superior anterior cingulate gyrus, posterior cingulate gyrus, left paracentral lobule, and **left inferior frontal gyrus**, inferior aspect	Typically reading and reading-impaired individuals	Cope et al. 2012
		Brain function—EEG	Occipitotemporal regions	Typically developing children	Su et al. 2014
*KIAA0319*	Cilia length, neural migration, axon growth	Cortical thickness	Left orbitofrontal region	Typically reading population	Eicher et al. 2016
		White matter volume	Left temporoparietal region; white matter tracts connecting the left **middle temporal gyrus** and **intraparietal lobule**	Typically reading children and young adults	Darki et al. 2012, 2014
		Brain function—fMRI	Superior temporal sulcus	Typically reading individuals	Pinel et al. 2012
*NRSN1*	Axon and dendrite growth	Gray matter volume	Right dorsal parieto-occipital cortex, left lateral occipital cortex, **visual word form area**	Typically reading and reading-impaired individuals	Skeide et al. 2016
		White matter volume	Left postcentral gyrus	Typically reading and reading-impaired individuals	Skeide et al. 2016

*Note:* Previous associations of dyslexia susceptibility genes *DNAAF4*, *DCDC2*, *KIAA0319*, and *NRSN1* with brain structure and brain function, based on which these genes were chosen as the genes of interest in the current study. The specific SNV: rs3743204 (in *DNAAF4*), rs793842 (in *DCDC2*), and rs6935076 (in *KIAA0319*) were chosen based on Darki et al. (2012, 2014), rs9461045 (in *KIAA319*) based on Eicher et al. (2016), and rs10946672 (in *NRSN1*) based on Braineac database. All these genes have been associated with brain structure in neurotypical individuals. Only *DCDC2* and *KIAA0319* have been associated with brain function using fMRI in previous studies.

**Fig. 1:**
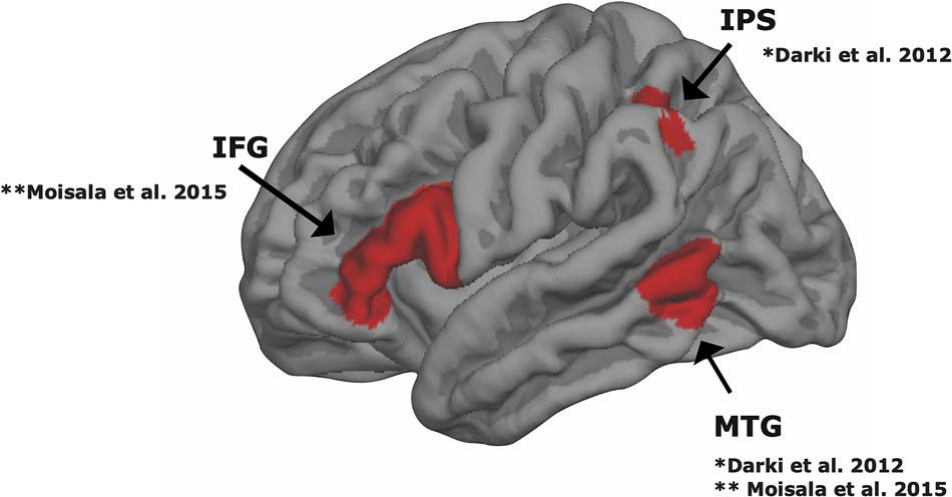
Lateral view of pial surface of the left hemisphere highlighting the three regions of interest (ROIs): IFG, MTG, and IPS. These ROIs were selected based on two previous studies: ^*^Darki et al. (2012) and ^**^Moisala et al. (2015). In Darki et al. (2012), white matter in the tracts connecting the left IPS and MTG was associated with variation in DD susceptibility genes. Moisala et al. reported increased brain activity in the same MTG region during processing of incongruent written and spoken sentences in comparison with congruent sentences. Moisala et al. (2015) reported a similar incongruency effect also in the left IFG. Moreover, brain function during reading in these three regions has been consistently associated with DD (Martin et al. 2016).

We used a region of interest (ROI)–based analysis on three predetermined regions: the left IFG, IPS, and MTG (see Fig. 1). The ROIs were selected based on previous studies. In Darki et al. (2012) DD susceptibility genes *DNAAF4*, *DCDC2*, and *KIAA0319* were associated with white matter tracts connecting the left MTG and IPS. Moisala et al. (2015) reported increased brain activity in an overlapping MTG region and the left IFG during the processing of incongruent written and spoken sentences. Notably, brain activity in all three ROIs has been associated with DD (Martin et al. 2016), but the current study is the first one exploring associations between DD susceptibility genes and brain function in these brain areas.

Our main preregistered (https://osf.io/7sehx) hypotheses concerning this study were as follows: (1) The SNVs rs3743204 (in *DNAAF4*), rs793842 (in *DCDC2*), rs10946672 (in *NRSN1*), rs6935076, and rs9461045 (in *KIAA0319*) are associated with hypoactivation in the left MTG, IFG, and IPS, and (2) variation within these genes contributes to differences in functional connectivity between the left MTG, IFG, and IPS. These hypotheses are based on previous studies showing hypoactivation (Kronbichler et al. 2006; Schulz et al. 2008; Devoto et al. 2022), weaker N400 event-related brain potential component (Schulz et al. 2008), and altered functional connectivity (Koyama et al. 2013) within the language network in individuals with DD in comparison with neurotypical participants. In addition, differences in white matter connections in language networks have been associated with the DD susceptibility genes targeted here (Darki et al. 2012, 2014) and reading ability (Vandermosten et al. 2016). Functional connectivity and psychophysiological interaction (PPI) analyses were included to provide a more comprehensive understanding of the brain networks involved in language processing. As we studied fluently reading individuals, possible differences in functional connectivity and PPI could help in clarifying the compensatory mechanisms helping individuals with genetic predisposition to DD to manage reading. In addition, we explored whether variation in task performance is associated with variation in these genes, considering that functional differences may either result from or influence linguistic performance.

We used the fMRI paradigm developed by Moisala et al. (2015), in which participants classify written or spoken sentences as semantically congruent or incongruent, in the presence or absence of distractor sentences in the other modality. This paradigm was chosen because Moisala et al. (2015) reported increased brain activity in neurotypical young adults to written and spoken incongruent sentences (in comparison with congruent sentences) in the left posterior MTG. The MTG regions was overlapping with the area where Darki et al. (2012) showed an association between white matter tracts and DD susceptibility genes. A similar incongruency effect was observed by Moisala et al. (2015) in the left IFG, which is also a core node of the language network and consistently associated with DD (Martin et al. 2016).

By focusing on fluently reading individuals with this genetic neuroimaging approach, we aimed to elucidate how genetic factors influence normal interindividual variability in language processing. Previous studies have reported associations between the genes targeted here and brain structure in fluently reading population (Meda et al. 2008; Darki et al. 2012, 2014; Eicher et al. 2016; Skeide et al. 2016), while the influence of these genes on brain function in fluent readers remains unclear. With the approach selected in the present study, we can hopefully get valuable insights into the primary causes leading to interindividual variability in reading skills.

## Materials and methods

### Ethics statement and preregistration

The participants’ suitability for fMRI scanning was screened following the standard procedure of the imaging site, the Advanced Magnetic Imaging Center at Aalto University, Finland. The experimental protocol was approved by the Ethics Committees of the Hospital District of Helsinki and Uusimaa, Finland. All participants gave written informed consent and were compensated for their time spent at the imaging site (€15/h). Written consent was obtained from the guardians when the participant was a minor. The study was preregistered and the preregistration is available online at the Open Science Framework (https://osf.io/kymjx). In the original preregistration, rs10946672 was incorrectly stated to be located within the gene *DCDC2*. In the corrected preregistration (https://osf.io/7sehx), it is located within the *NRSN1* gene, which is adjacent to *DCDC2* at the *DYX2* locus (Eicher et al. 2014). In the present study, we focused on two of the main research questions posed in the preregistration: (1) Does variation in dyslexia susceptibility genes *DNAAF4*, *DCDC2*, *NRSN1*, and *KIAA0319* explain variation in brain activity in the MTG, IFG, and IPS, and (2) does variation in dyslexia susceptibility genes *DNAAF4*, *DCDC2*, *NRSN1*, and *KIAA0319* explain functional connectivity with seed points in MTG, IFG, and IPS?

### Participants

Functional MRI data, together with structural magnetic resonance imaging (sMRI) data, were collected from 267 participants (124 females). The present study sample partly overlaps with studies using the same paradigm on attention-related brain functions in large groups of 13–24-year-old neurotypical participants (Moisala et al. 2016, 2018). Those with excessive head motion (mean framewise displacement > 0.5 mm) or anatomical anomalies that affected co-registration were excluded from the analysis.

The participants were 13–25-year-old (mean, females: 16.4 years; males: 17.0 years) native Finnish speakers with self-reported normal hearing and normal or corrected-to-normal vision. They neither had a self-reported history of psychiatric or neurological illnesses, nor learning disabilities at the time of the measurement. The participants were either university students, or middle- or high-school students screened based on their academic performance. Depending on the sample cohort, the school-aged participants had either self-reported grade point average (GPA) >7 (2013–2015), or math grade ≥7 (2019–2020), on the 4-to-10 point scale system used in Finnish schools. This difference in screening arose because the participants of the latter measurements also participated in another study focusing on arithmetic processing (Ylinen et al. in preparation).

### Stimuli

**Fig. 2:**
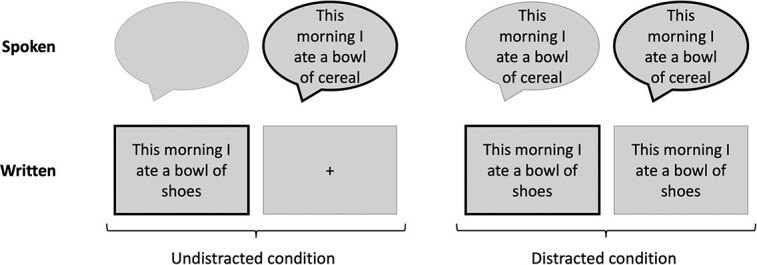
A schematic illustration of the experimental protocol used in the fMRI measurements. Black outlines indicate the modality the participants were to attend to. The same sentence was never used twice, but the same sentence stem was used in incongruent and congruent sentences, although in different runs.

The stimuli consisted of Finnish semantically congruent and incongruent sentences (e.g. *This morning I ate a bowl of cereal/shoes*), presented either in written or spoken form (see Fig. 2). The congruent sentences were simple phrases about everyday subjects. The incongruent sentences were created by replacing the last word of a congruent sentence with a semantically incongruent but syntactically plausible word.

During the fMRI measurements, each sentence was presented on a screen and was visible for 2.5 s. The last word of each sentence was initially concealed with a string of letters x (equal in length to the original word) for the first 2 s of the trial. The auditory sentences were spoken by a female native Finnish speaker. Each sentence lasted for ~2.5 s. More details about the stimuli can be found in the Supplementary Material (see Suppl. 4.1) and the stimuli creation from Moisala et al. (2016) reporting attention-related brain activity from a partly overlapping group of participants.

### Functional MRI procedure

The fMRI measurements in the two cohorts, with distinct sets of participants, were conducted over two different time periods (2013–2015 and 2020–2021). During 2020–2021, the participants were presented with eight different experimental conditions, defined by task (reading, listening), distractor (present, absent), and sentence congruence (congruent, incongruent) (Fig. 2). Four different combinations of tasks and distractors (2 tasks × 2 distractors) were presented in blocks and the congruent and incongruent sentences were presented within these blocks in a random order. The four task blocks included unimodal and distracted auditory and unimodal and distracted visual blocks. Unimodal blocks only had written or spoken sentences and distracted blocks both.

The spoken or written distractor sentences were presented in the task-irrelevant modality. The distractor sentences were randomly either congruent or incongruent, semantically unrelated to the attended sentence, and presented so that the last word in the concurrent attended and distractor sentences occurred at the same time. The participants were asked to ignore the distractor sentences and focus their attention on the sentences in the task-relevant modality. During the unimodal block and a rest block, the participants were asked to fix their gaze on a fixation cross in the center of the screen.

In the beginning of each block, written instructions for the task at hand were presented for 3.5 s. Each task block consisted of 12 sentences or pairs of written and spoken sentences (distracted condition). Half of the sentences were congruent and the other half incongruent, the order of congruent and incongruent sentences being randomized. Each sentence was followed by a 1-s response window, during which a question mark (size 1.4° × 1.0°) was presented in the center of the screen. The participants were instructed to respond by pressing a button with their right index or middle finger, indicating whether the attended sentence was congruent or incongruent, respectively. At the end of each block, feedback (i.e. the percentage of correct responses within the block) was presented for 2 s, followed by a 4-s rest before the next block.

In each functional run, all four different task blocks and a 40-s rest block were presented once. The order of the task blocks during each run was randomized, and the rest block was always between the second and third task blocks. Data during three functional runs were collected from each participant. Before the experiment, the participants practiced all tasks outside the scanner. Sentences presented in the practice trial were not used in the experiment.

During the 2013–2015 measurements, five additional task blocks not used in the current study were included (see Suppl. 4.1). For further details, see the Supplementary Material (Suppl. 4.2) and Moisala et al. (2016, 2018).

### Data acquisition

Brain imaging was conducted at the Advanced Magnetic Imaging Centre at Aalto University (Espoo, Finland). The imaging was carried out using a 3-T MAGNETOM Skyra whole-body scanner (Siemens Healthcare, Erlangen, Germany) using a 20- or 30-channel head coil, depending on the measurement period (2013–2015 and 2020–2021, respectively).

During fMRI data collection, functional echo planar images were acquired with an imaging area consisting of 43/56 (2013–2015/2020–2021 measurements) continuous oblique axial slices (TR 2500 ms/1300 ms, TE 32 ms/41 ms, flip angle 75°/65°, voxel matrix 64 × 64/96 × 96 mm, field of view 200 mm/240 mm, slice thickness 3.0 mm/2.5 mm, in-plane resolution 3 mm × 3 mm × 3 mm/2.5 mm × 2.5 mm × 2.5 mm), and three functional runs of 222/202 volumes were acquired from each participant. Thus, depending on the measurement period, a total of 666 or 606 functional volumes were obtained per session (session duration was ~27 or 13 min).

During sMRI data collection, high-resolution anatomical images consisting of 176 slices (TR 2530 ms, TE 3.3 ms, flip angle 7°, voxel matrix 256 × 256, field of view 256 mm, slice thickness 1 mm, in-plane resolution 1 mm × 1 mm × 1 mm) were acquired. The imaging time for the T1-weighted images was ~6 min.

Genetic data were collected in 2019–2021. Participants who took part in the MRI measurements during 2013–2015 were contacted and genetic samples were collected from those consenting. Those who participated in 2020–2021 gave, upon consent, the genetic samples during the same session as the measurement. Consent for genetic sampling was collected from guardians when the participant was a minor. Adult participants were given a choice to give the genetic sample as a blood or a saliva sample, whereas minors only gave saliva samples. Altogether, 93 blood samples and 102 saliva samples were collected.

Genetic data were acquired from 186 participants. Depending on the SNV and the genotyping success rate, the analyses were conducted on 179 (rs3743204 in *DNAAF4*, *rs*10946672 in *NRSN1*, rs9461045 in *KIAA0319*), 171 (rs793842 in *DCDC2*), or 178 (rs6935076 in *KIAA0319*) participants, respectively. Genotyping was performed at a certified core facility at Karolinska University Hospital using the iPLEX (Agena) platform (https://www.maf.ki.se/snp-genotyping-agena/). The genotype group sizes in our sample were consistent with the minor allele frequencies reported in the National Center for Biotechnology Information and are reported in Supplementary Table 1. Genetic samples were pseudonymized and will be stored at the Folkhälsan Research Centre (Helsinki, Finland) for 10 years following the last publication based on the samples.

### Data analysis

#### Functional MRI preprocessing and analysis

Task blocks where the percentage of correct responses was more than 3 SD below the average across participants (55 blocks altogether) were removed as the low performance might be due to performing a wrong task or not performing any task.

The fMRIPrep pipeline (Esteban et al. 2019) was used to preprocess the fMRI data (see Suppl. 4.3), resulting in co-registered preprocessed data on the fsaverage surface. ICA-AROMA was used to denoise the data for functional connectivity analyses (Pruim et al. 2015) (see Suppl. 4.3).

First-level analyses were conducted using FSL and included the eight conditions as regressors, as well as nuisance regressors (see Suppl. 4.4). Contrasts were defined for each condition separately, as well as a contrast for all unimodal reading and listening conditions separately. Group-level whole-brain fMRI analysis was conducted using Freesurfer software (see Suppl. 4.4).

For ROI analyses, three ROIs in the left hemisphere were created in Freesurfer’s fsaverage space (Fig. 1). A frontal ROI in the IFG was selected based on across-participant data from the study by Moisala et al. (2015) and anatomically defined using Freesurfer’s cortical annotations. The IFG ROI included the pars opercularis and pars triangularis. Temporal and parietal ROIs in the MTG and IPS were defined using coordinates reported by Darki et al. (2012) and created by drawing a circular ROI with a radius 12.5 mm around the cortical coordinates. The percent signal changes were thereafter determined using FSL’s Featquery tool, which converts the extracted signal into percent signal change compared to silent rest. The values were extracted within each ROI and averaged across the three runs separately for each condition, except for four participants, for whom only two runs were used, due to technical errors or too much framewise displacement during one of their runs.

For the functional connectivity analyses, preprocessed timeseries data were extracted from each ROI (IFG, MTG, IPS in the left hemisphere) separately for each run. Within each ROI, the timeseries from the three, or two if only two runs for a participant were used in the analysis, runs were effectively concatenated (see Cho et al. 2021). A 3 × 3 correlation matrix between the concatenated timeseries from the three ROIs was created.

In the PPI analysis, separate general linear models with the psychological regressor for the contrast of interest (all eight task conditions vs. rest) with a physiological regressor (mean timeseries of the seed ROI) and a PPI regressor (interaction between the psychological and physiological regressors) were created. In addition, regressors for the task blocks not included in this study (see Suppl. 4.2) and nuisance regressors (see Suppl. 4.4) were added to the model. PPI beta weights were extracted using the ROI masks (see above) for the subsequent statistical analysis.

#### Analysis of behavioral data

The total percentage of correct responses per task was calculated from the behavioral data. As in the fMRI analysis, blocks with the percentage of correct answers >3 SD below the average across the participants were removed from the analysis.

#### Statistical analysis

First, analyses of variance (ANOVAs) with the same structure for the ROI fMRI, functional connectivity, and PPI were conducted. For the ROI fMRI analysis, the percent signal change in our three ROIs (MTG, IPS, IFG in the left hemisphere) and for functional connectivity as well as the PPI analyses, the ROI–ROI correlation (MTG–IFG, MTG–IPS, and IPS–IFG in the left hemisphere) were used as the dependent variables. Separate models were created for each SNV (*DNAAF4*: rs3743204, *NRSN1*: rs10946672, *DCDC2*: rs793842, and *KIAA0319*: rs6935076, rs9461045) where the allelic group was the between-subject factor and the within-subject factors were always task (reading, listening), congruence (congruent, incongruent), and distractor (distractor, no distractor). In rs3743204 (*DNAAF4*), rs10946672 (*NRSN1*), and rs6935076 (*KIAA0319*), the smaller homozygote group comprised < 7 participants and was combined with the heterozygote group for the analysis. As these are common variations, it is likely that the effects of alleles are additive, and combining two groups for the statistical analysis therefore increases the power of the analysis. In contrast to the preregistration, we did not explore for quadratic trends, as it would have been possible only in two SNVs, where the three allele groups could be studied separately.

All analyses included age, sex, and the sample cohort as the between-subject covariates. To decrease the number of conducted comparisons, we decided to run only analyses with covariates, although in the preregistration, we had planned to perform all analyses with and without covariates. The sample cohort was added as a covariate, as the fMRI measurement parameters varied between the two measurement periods. *T*-tests for age and chi-squared tests for gender and sample cohort were conducted for each gene. The results, including group distribution, can be found in the Supplementary Material (see Supplementary Tables 2–6). The effect of age group (13–15 years, 16–18 years, 19–25 years) on brain activity in the three ROIs was explored using ANOVA, and the results are shown in the Supplementary Material (see Suppl. Fig. 1).

The behavioral results were studied using mixed ANOVAs as for the fMRI data, separately for all five SNVs, with the percentage of correct responses as a dependent variable.

For the models with significant gene effects, we used log-likelihood tests to compare full models, with all effects (including gene effects) to null models with all effects excluding the gene effects. This provides an estimate for whether the inclusion of the gene main effects and interactions significantly improves the model in comparison to the null model. This was performed in the following manner: First, we defined a linear mixed model with REML estimation with all explanatory variables, as our full model, and made sure that it exactly replicated our mixed ANOVA results. Then, we changed the estimation to ML to get an estimate for the likelihood of the full model, as ML allows for model comparisons. Thereafter, we removed the genetic effects and estimated the likelihood for the null model (i.e. without the gene effects). Finally, log-likelihood ratios were calculated between the full and the null model. The likelihood ratio was then tested using the chi-squared (χ^2^) test to see if adding genetic effects significantly improves the null model, with the equation (2(ln(null model likelihood) − ln(full model likelihood)) (as described in Huelsenbeck and Crandall 1997).

As the focus of this study was on the effects of genetic variation, only effects with the gene factor were assessed. Greenhouse–Geisser correction was used where appropriate, and the corrected degrees of freedom are reported. All statistical analyses were conducted using either R statistical software (version 4.0.2.; R Foundation for Statistical Computing, Vienna, Austria) or JASP (version 0.16.4).

## Results

### Whole-brain analysis

**Fig. 3:**
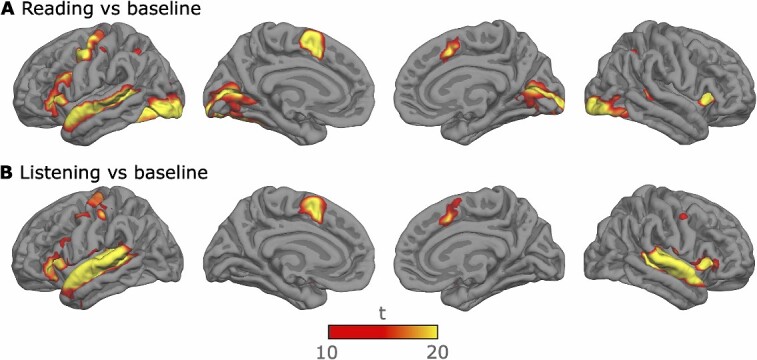
Significant clusters for the effects of reading **A**) and listening **B**) in relation to the resting block baseline. **T*-v*alues indicate the strength and statistical significance of activation, with higher *t*-values indicating more robust neural responses to the respective stimuli.

Figure 3 shows brain activity during the listening and reading tasks. Language processing during both tasks modulated brain activity in the IFG and superior temporal (auditory) and inferior parietal cortices. Moreover, during both tasks, activity apparently associated with motor responses was observed in the left motor and premotor cortices, as well as in the medial supplementary motor area. In addition, the occipital (visual) cortex was strongly activated during the reading task.

### Behavioral

**Fig. 4:**
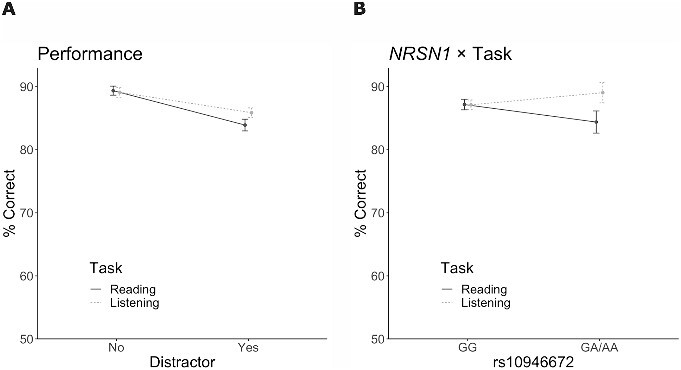
Task performance (% of correct responses). The *x*-axis is anchored at 50% corresponding to chance level performance. **A**) Task performance for congruent and incongruent sentences across the four task conditions. **B**) The effect of rs10946672 (*NRSN1*) on performance during reading and listening tasks (data for unimodal and distracted conditions, incongruent and congruent attended sentences combined). The plot illustrates the percentage of correct responses in classifying attended sentences as incongruent or congruent, comparing individuals with GG genotype (*n* = 145) and GA/AA genotypes (*n* = 34). Error bars denote the standard error of the mean (SEM).

Overall, the participants performed well in the present fast-paced reading and listening tasks. Performance was better in the unimodal than in the distracted reading and listening conditions (Task × Distractor interaction effect *F*(1,176) = 5.69, η^2^ = 0.002, *P* = 0.018; Fig. 4A). For rs10946672 (*NRSN1*), there was a significant gene × task interaction (*F*(1,175) = 4.36, η^2^ = 0.005, *P* = 0.038; Fig. 4B). This interaction was explained by worse performance in the reading task in those carrying the less common A allele than in the common GG genotype. The A-allele carriers performed better during the listening task than during the reading task, whereas individuals with the GG genotype performed at the same level in both conditions. The log-likelihood test showed that adding gene effects to the linear mixed null model including all within-subject effects and covariates improved the model significantly (χ^2^(8) = 16, *P* = 0.042). No other significant gene effects were found in the behavioral results.

### Functional MRI ROI analysis

#### DNAAF4 gene

**Fig. 5:**
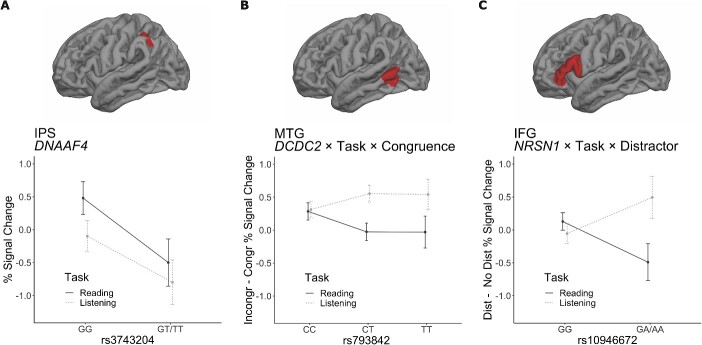
The three ROIs, IPS (**A**), MTG (**B**), and IFG (**C**), on the lateral view of the pial surface of the left hemisphere**. A**) The effect of rs3743204 (*DNAAF4*) on brain activity in the IPS ROI: % signal change averaged across all conditions in relation to resting task baseline. The plot compares brain activity in individuals with GG (*n* = 121) and GT/TT (*n* = 58) genotypes. **B**) The interaction effect of rs793842 (*DCDC2*) × task × congruence. The plot displays brain activity in the MTG between individuals with CC (*n* = 74), CT (*n* = 75), and TT (*n* = 22) genotypes. The congruency (Incongr—Congr) % signal change is calculated by subtracting the % signal change during congruent sentences from that change during incongruent sentences. **C**) The interaction effect of rs10946672 (*NRSN1*) × task × distractor. The plot displays brain activity in the IFG between individuals with GG genotype (*n* = 144) and GA/AA genotypes (*n* = 35). The distractor (Dist—No Dist) % signal change is calculated by subtracting the % signal change in the unimodal condition from that in the distracted condition. Error bars denote the standard error of the mean (±SEM).

A significant main effect of rs3743204 was found in the IPS ROI (*F*(1,174) = 4.56, η^2^ = 0.016, *P* = 0.034; Fig. 5A). In T-allele carriers, unlike in the more common GG genotype, there was deactivation in the IPS ROI during the language tasks (reading and listening) compared to the task baseline. In accordance, the log-likelihood test showed that adding gene effects to the null model including all within-subject effects and covariates improved the model significantly (χ^2^(8) = 26, *P* = 0.001).

#### DCDC2 gene

A significant gene × task × congruence interaction was found for rs793842 (*DCDC2*) in the MTG ROI (*F*(2,165) = 3.29, η^2^ = 0.001, *P* = 0.04; Fig. 5B) due to different congruency effects in the listening and reading tasks in individuals carrying the T allele. During the listening task, incongruent sentences resulted in increased brain activity in the MTG, whereas during the reading task no such incongruency effect occurred in T-allele carriers. For individuals with the CC genotype, incongruent sentences were associated with increased brain activity in the MTG during both reading and listening tasks. Again, the log-likelihood test showed that adding gene effects to the null model improved the model significantly (χ^2^(16) = 202, *P* < 0.001).

#### NRSN1 gene

For rs10946672 (*NRSN1*), there was a significant gene × task × distractor interaction in the IFG (*F*(1,174) = 6.12, η^2^ = 0.003, *P* = 0.014; Fig. 5C) due to different distractor effects during the listening and reading tasks in the A-allele carriers. During the reading task, brain activity in the IFG increased during the distracted condition, whereas during the listening task, the text distractor was associated with decreased IFG activity. In individuals with the more common GG genotype, brain activity did not differ significantly between different conditions. The log-likelihood test provided further support for this effect by showing that adding gene effects improved the null model significantly (χ^2^(8) = 16, *P* = 0.042).

#### KIAA0319 gene

No associations between the SNVs rs6935076 or rs9461045 (*KIAA0319*) and brain activity in any ROI during the present tasks were found (*F* < 2.72, *P* > 0.101, in all cases).

### Functional MRI functional connectivity

No significant gene effects were found in the functional connectivity (*F* < 3.45 *P* > 0.065) and PPI analysis (*F* < 1.72, *P* > 0.18, in all cases).

## Discussion

We studied the relationship between genes previously associated with DD and structural differences in the brain language network with brain activity during language processing. We focused on the DD susceptibility genes *DNAAF4*, *DCDC2*, *NRSN1*, and *KIAA0319* in a cohort of fluently reading adolescents and young adults. By combining neuroimaging and genetic data, we revealed how variation in three SNVs previously associated with structural brain alterations is also associated with brain function. We studied brain activity during a reading task that has been shown to correlate with standardized clinical measures of reading fluency (Christodoulou et al. 2014), providing insights into the neural mechanisms underlying DD susceptibility. With an analogous listening task, we were able to evaluate whether possible anomalies associated with the studied DD susceptibility genes were specific to reading or extended to general linguistic processing.

Our findings provide compelling evidence that allelic variation in *DNAAF4*, *DCDC2*, and *NRSN1* is associated with brain activity in key language processing regions: the left IFG, MTG, and IPS. It is important to note that the specific functions of the SNVs studied here are not known. For instance, rs3743204 is in strong linkage disequilibrium with rs3743205, which affects an Elk-1 transcription factor binding site within *DNAAF4* (Taipale et al. 2003), but this has not been studied in further detail. No studies have focused on the functional aspects of the remaining SNVs. Rs3743204, rs793842, and rs10946672 have low Combined Annotation Dependent Depletion scores suggesting an unlikely strong direct functional effect. Further supporting this, in rs9461045 opposing alleles correlate with dyslexia risk in different populations (Shao et al. 2016). Therefore, it is likely that the SNVs are not functional themselves, but rather linked to the actual causal variants. Consequently, the direction of the genetic effects reported in this study is not the primary focus as different populations may exhibit varying alleles linked to susceptibility phenotypes.

In previous studies, Darki et al. (2012, 2014) demonstrated an association of *DNAAF4* and *DCDC2* with white matter volume within the left MTG and IPS. To our knowledge, among the genes targeted in the present study, only *DCDC2* has been previously associated with brain function using fMRI, as reported by Cope et al. (2012) in individuals with reading disability. The associations in the present study were observed during tasks involving reading or listening to incongruent and congruent sentences in individuals with no reading difficulties. The participants achieved a high level of accuracy in all task conditions, suggesting that the observed genetic associations with brain activity may not be indicative of pre-existing language-related impairments. Previous neuroimaging studies have shown hyperactivation in right frontal brain regions in individuals with DD (Shaywitz et al. 2003; Hoeft et al. 2007, 2011; Yu et al. 2018), which has been suggested to serve as a compensatory mechanism for phonological difficulties (Hancock et al. 2017). In the current study, the detected changes in brain activity in the absence of behavioral differences may also signify compensatory processes utilized by individuals with a genetic predisposition to DD. These strategies could enable a typical level of performance in linguistic tasks despite potential genetic predisposition to DD.

Variation in *NRSN1* had a significant effect on task performance. Individuals carrying the A allele in rs10946672 performed worse in the reading task than the more common GG genotype group, indicating that genetic predisposition to DD influenced performance in rapid reading. This is consistent with the idea that DD represents the extreme end of a spectrum of reading abilities, a notion further supported by recent twin studies (for a review, see Erbeli et al. 2022). Erbeli et al. (2018) demonstrated that genetic factors influence reading skills across the continuum of reading abilities. Furthermore, Lind et al. (2010) showed that variation in *DCDC2* is associated with reading abilities in the general population. These studies, together with the present results, suggest that genetic predisposition and its association with reading skills are observable even among well-performing individuals. Interestingly, the A-allele carriers outperformed the GG genotype group in the listening task. These findings could be explained by a compensatory mechanism or an alternative processing strategy that relies more on auditory cues to overcome subtle difficulties in reading and phonological processing.

Consistent with our behavioral findings regarding *NRSN1*, individuals carrying the A allele in rs10946672 showed decreased brain activity in the left IFG during distracted reading, but not during distracted listening. Skeide et al. (2016) reported that gray matter changes in the VWFA, which were associated with variation in *NRSN1*, were predictive of reading abilities. However, to our knowledge, the present study is the first to investigate the association of *NRSN1* with brain function. The left IFG is known to be important for semantic processing of written sentences (Hagoort et al. 2004). Reading skills and DD have consistently been linked to brain activity in the left IFG during reading tasks (Schulz et al. 2008; Jobard et al. 2011; Christodoulou et al. 2014; Martin et al. 2016), as well as differences in white matter volume in frontal areas (Vandermosten et al. 2012). The observed effect of *NRSN1* on IFG activity may indicate that individuals carrying the allele associated with DD susceptibility have a reduced ability to focus on written sentences, resulting in a decreased IFG response when presented with spoken distractor sentences.

In the left IPS, brain activity was associated with variation in *DNAAF4*, suggesting distinct neural responses during language processing among individuals with genetic predisposition to DD. Specifically, those carrying the T allele in rs3743204 showed decreased brain activity in the left IPS across all the conditions. This aligns with prior research by Darki et al. (2012) reporting reduced white matter volume within the corresponding region in T-allele carriers of the same SNV. While the IPS is typically considered part of the default mode network, exhibiting decreased activity during active tasks compared to resting conditions (Raichle 2015), reading and particularly verbal short-term memory tasks have consistently been shown to elicit increased activity the left IPS (Majerus et al. 2006; Fujimaki et al. 2009; Ossmy et al. 2014; Majerus and Cowan 2016). Our study integrates structural findings from imaging genetics with functional studies without genetic data, emphasizing the direct relationship between DD susceptibility and reduced IPS activity. This strongly supports the key role of the left IPS in reading and language processing.

In the left MTG, we observed a significant interaction of *DCDC2*, task, and congruence. Prior research by Moisala et al. (2015) demonstrated increased brain activity in this region during reading and listening to incongruent sentences. In the current study, individuals with T allele in rs793842 did not display such incongruency effect in the left MTG during reading. However, during the listening task, the T-allele carriers exhibited an incongruency effect. In contrast, individuals with the more common CC genotype showed increased MTG activity in response to both incongruent written and spoken sentences. *DCDC2* has previously been associated with white matter structure in the left MTG (Darki et al. 2012, 2014) as well as gray matter volume in the left temporal areas in non-DD individuals (Meda et al. 2008). DD, in turn, has consistently been associated with reduced activity in the left MTG (Paulesu et al. 2001; Mccandliss and Noble 2003; Richlan et al. 2011; Martin et al. 2016). Our findings add to these studies by showing an association of *DCDC2* and left MTG function in typically reading individuals. Specifically, as our findings concerning *NRSN1*, the effect of *DCDC2* was specific to conditions while in *DNAAF4* the differences in brain activity between the two genotype groups were observed during both listening and reading. This suggests potential modality-specific and modality-independent linguistic effects associated with the here targeted DD susceptibility genes.

In previous research, intrinsic functional connectivity between the left IPS and left middle frontal gyrus has been lower in participants with DD when compared to typically developing children (Koyama et al. 2013). However, contrary to our hypothesis based on previous associations of *DNAAF4*, *DCDC2*, and *KIAA0319* with white matter volume in language networks (Darki et al. 2012, 2014), we did not find any genetic effects in the functional connectivity and PPI connectivity analyses. It is possible that the genetic influences on brain functional connectivity are more subtle and become evident only when the criteria for DD are met. The normal functional connectivity in reading networks among individuals with genetic predisposition to DD could offset the distinct activity patterns in reading-related regions. For example, Smirnov et al. (2014) reported that during sentence comprehension tasks, the activity of the left IFG was decreased as its functional connectivity with temporoparietal brain regions was strengthened. Normal functional connectivity might compensate for the weaker IFG activity in those with genetic predisposition to DD. Despite the lack of genetic effects on functional connectivity in the current study, it might be valuable that future functional imaging-genetics studies on reading and related difficulties would examine this issue more closely, as such effects could be affected by the specific experimental tasks being used.

In previous studies, *KIAA0319* has been associated with temporoparietal white matter volume (Darki et al. 2012) and the locus containing *KIAA0319* with brain activity of the superior temporal sulcus during sentence reading (Pinel et al. 2012). In addition, *KIAA0319* has been associated with reading skills both in the dyslexic and neurotypical population (Scerri et al. 2011), supporting our hypothesis that *KIAA0319* could impact brain activity in our data consisting of neurotypical individuals. However, we did not find any significant effects of the two SNVs in *KIAA0319* in neither behavioral nor fMRI results. This implies that possible *KIAA0319*-related structural changes in the present study could have been functionally compensated.

Like most of the imaging genetics studies on DD, the present study is cross-sectional and involved an age group that has years of reading exposure. Therefore, we cannot be sure if the observed neuroanatomical and neurofunctional variability associated with certain genetic variants predates reading onset or is a consequence of years of poor reading. However, as the sample of the present study consisted of fluently reading individuals with no history of reading disabilities or any other learning deficits, our results support the view that the observed genetic effects are the primary cause of the differences seen in brain function. To further clarify this issue, longitudinal studies where the data collection begins already prior to reading exposure should be conducted in the future.

We studied individuals between the ages of 13 and 24. This is a phase of significant developmental changes in the brain, encompassing synaptic pruning (Petanjek et al. 2011), dendritic growth (Petanjek et al. 2019), and changes in the dopaminergic system (Islam et al. 2021; Wahlstrom et al. 2010). To account for the impact of these developmental changes on our participants, age was included as a covariate in all our analyses. However, our sample size was insufficient to examine the genetic effects separately in different age groups. Future studies should delve into this aspect to understand how genetic factors influence these crucial developmental processes that underlie cognitive functions.

## Limitations

While the present study provides important evidence of links between DD susceptibility genes and brain function, there are some limitations worth mentioning. First, we only included well-performing individuals with an above-average academic GPA or mathematics grade, or already with some university education. Therefore, our study sample is not fully representative of the typically reading population. Second, it is important to note that the gene effects reported are fairly small. In our preregistration, we stated that we would employ either false discovery rate or family-wise error rate correction to address multiple comparisons where appropriate. However, while conducting the study, we realized that this correction would be too stringent and might inflate type II errors. Therefore, instead, we used log-likelihood tests (see Results 3.1 and 3.2) to test whether the model with gene effects is more likely than the model without them given the data.

## Conclusion

The present findings support the view that difficulties in reading may have multiple developmental trajectories driven by various genetic and environmental factors. As proposed by O’Brien and Yeatman (2020), DD might not be attributable to a single core deficit, but rather be due to various dysfunctions affecting neurolinguistic development. Apparently, some individuals with genetic predisposition to DD can develop behavioral and neural compensatory mechanisms, resulting in distinct brain activity patterns as seen in our results. Alternatively, there might be subclinical intraindividual variability in neurocognitive strength profiles that may remain unnoticed: For instance, at the same time when risk allele carriers may have difficulties in reading, they might have strengths in listening to speech. Notably, brain activity patterns associated with linguistic tasks are modulated by variations in different DD susceptibility genes in a specific manner, further underscoring their role in brain functions or dysfunction associated with DD. Our results highlight the complex and multifactorial nature of DD as well as the relevance of these DD susceptibility genes in language-related brain function.

## Supplementary Material

Rinne_et_al_2024_supplementary_material_bhae144
