# Supplementary material for: Developmental dyslexia susceptibility genes DNAAF4, DCDC2, and NRSN1 are associated with brain function in fluently reading adolescents and young adults
Source: Cereb Cortex. 2024 Apr 12;34(4):bhae144. doi: 10.1093/cercor/bhae144 (PMC11014888; doi:10.1093/cercor/bhae144)
Supplement: Rinne_et_al_2024_supplementary_material_bhae144 [file rinne_et_al_2024_supplementary_material_bhae144.doc]

**Supplementary information**

1. Supplementary Figure 1.
2. Supplementary Table 1.
3. Demographic and group distribution tables

*3.1 Supplementary Table 2*

*3.2 Supplementary Table 3*

*3.3 Supplementary Table 4*

*3.4 Supplementary Table 5*

*3.5 Supplementary Table 6*

1. Supplementary Methods

*4.1 Stimuli*

*4.2 fMRI procedure*

*4.3 fMRI preprocessing*

*4.4 fMRI analyses*

*References*


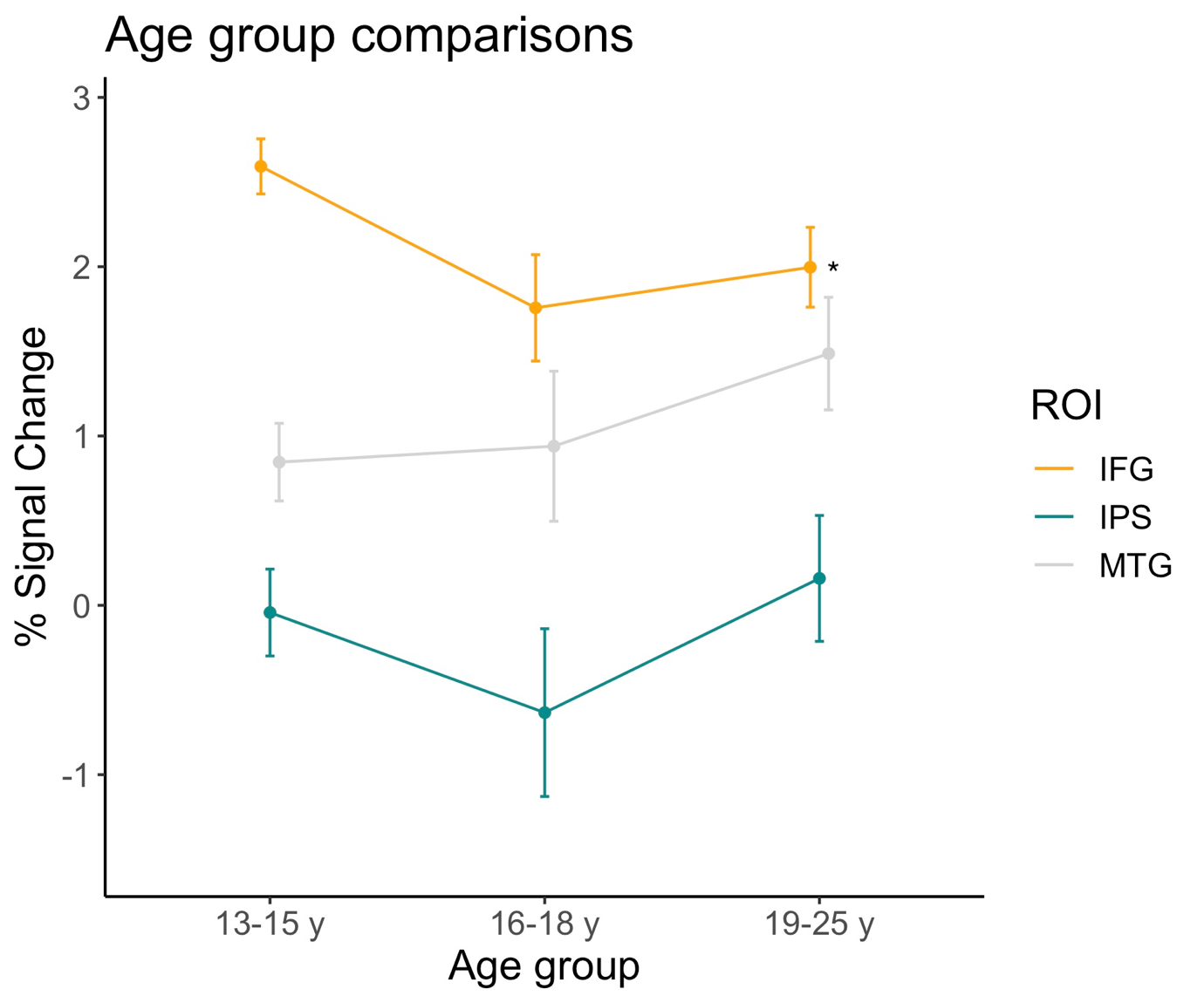


1. **Supplementary Figure 1.** The main effect of age group (13-15 yrs., 16-18 yrs., 19-25 yrs.) on brain activity in three ROIs: the left inferior frontal gyrus (IFG) (orange), the left intraparietal sulcus (IPS) (green) and the left middle temporal gyrus (MTG) (grey). The y axis shows the % signal change averaged over all conditions in relation to resting task baseline. The main effect of age was significant only in the IFG (F(2,147) = 3.30, η2 = 0.022, p = 0.039). Brain activaty in the IFG across all the conditions (reading and listening, distracted and undistracted, incongruent and congruent) was higher in the youngest age group.
2. Supplementary table 1.

| ***Table 1*.** *Distribution of alleles in candidate genes.* | | | | | |
| --- | --- | --- | --- | --- | --- |
| Gene | SNV | Genotypes (N) | | | MAF (%) |
| *DNAAF4* | rs3743204 | GG (121) | GT (52) | TT (6) | (T) 0.19 |
| *DCDC2* | rs793842 | CC (74) | CT (75) | TT (22) | (T) 0.41 |
| *NRSN1* | rs10946672 | GG (145) | GA (30) | AA (4) | (A) 0.29 |
| *KIAA0319* | rs6935076 | CC (67) | CT (94) | TT (17) | (T) 0.09 |
| *KIAA0319* | rs9461045 | CC (137) | CT (37) | TT (5) | (T) 0.18 |
| *Note.* The minor allele frequencies (MAF) are based on European ancestry frequencies reported in the National Center for Biotechnology Information. dbSNP: Short Genetic Variations. [Accessed 2024-02-09]. https://www.ncbi.nlm.nih.gov/snp/ | | | | | |

1. Demographic and group distribution tables

| ***Table 2.*** *Demographic and Group Distribution for Gene DNAAF4 rs3743204* | | | | |
| --- | --- | --- | --- | --- |
| Characteristics | GG  (*N* = 121) | GT/TT  (*N* = 58) | Test statistic (*df*) | *p* |
| Age, *M (SD)* | 16.8 (3.54) | 16.5 (3.36) | *F* (1,177) *=* 0.26 | .61 |
| Gender, male / female | 65 / 56 | 27 / 31 | χ² (1) = 0.54 | .46 |
| Sample Cohort (2013-2015 / 2019-2020) | 68 / 53 | 30 / 28 | χ² (1) = 0.16 | .69 |
| *Note. df* = degrees of freedom, *M* = mean, *SD* = standard deviation. | | | | |

| ***Table 3.***  *Demographic and Group Distribution for Gene DCDC2 rs793842* | | | | |  |
| --- | --- | --- | --- | --- | --- |
| Characteristics | CC  (*N* = 74) | CT  (*N* = 75) | TT  (*N* = 22) | Test statistic (*df*) | *p* |
| Age, *M (SD)* | 16.9 (3.70) | 17.1 (3.51) | 16.0 (2.83) | *F* (2,168) *=* 0.88 | .42 |
| Gender, male / female | 40 / 34 | 34 / 41 | 13 / 9 | χ² (2) = 1.82 | .40 |
| Sample Cohort (2013-2015 / 2019-2020) | 39 / 35 | 48 / 27 | 11 / 11 | χ² (4) = 2.49 | .29 |
| *Note. df* = degrees of freedom, *M* = mean, *SD* = standard deviation. | | | | |  |

| ***Table 4.*** *Demographic and Group Distribution for Gene NRSN1 rs10946672* | | | | |
| --- | --- | --- | --- | --- |
| Characteristics | GG  (*N* = 145) | GA/AA  (*N* = 34) | Test statistic (*df*) | *p* |
| Age, *M (SD)* | 16.3 (4.25) | 18.4 (3.16) | *F* (1,177) *=* 10.12 | .002 |
| Gender, male / female | 74 / 71 | 18 / 16 | χ² (1) = 9.19×10^-5^ | .99 |
| Sample Cohort (2013-2015 / 2019-2020) | 76 / 69 | 22 / 12 | χ² (1) = 1.22 | .27 |
| *Note. df* = degrees of freedom, *M* = mean, *SD* = standard deviation. | | | | |

| ***Table 5.*** *Demographic and Group Distribution for Gene* *KIAA0319 rs6935076* | | | | |  | |
| --- | --- | --- | --- | --- | --- | --- |
| Characteristics | CC  (*N* = 67) | CT  (*N* = 94) | TT  (*N* = 17) | Test statistic (*df*) | *p* | |
| Age, *M (SD)* | 16.7 (3.53) | 17.0 (3.57) | 15.7 (2.65) | *F* (2,175) *=* 1.11 | .33 | |
| Gender, male / female | 39 / 28 | 49 / 45 | 4/13 | χ² (2) = 6.55 | .04 |  |
| Sample Cohort (2013-2015 / 2019-2020) | 39 / 28 | 51 / 43 | 8/9 | χ² (2) = 0.73 | .69 | |
| *Note. df* = degrees of freedom, *M* = mean, *SD* = standard deviation. | | | | | | |

| ***Table 6.*** *Demographic and Group Distribution for Gene KIAA0319 rs9461045* | | | | |
| --- | --- | --- | --- | --- |
| Characteristics | CC  (*N* = 137) | CT/TT  (*N* = 42) | Test statistic (*df*) | *p* |
| Age, *M (SD)* | 16.8 (3.52) | 16.5 (3.36) | *F* (1,177) *=* 0.16 | .69 |
| Gender, male / female | 69 / 68 | 23 / 19 | χ² (1) = 0.10 | .75 |
| Sample Cohort (2013-2015 / 2019-2020) | 78 / 59 | 20 / 22 | χ² (1) = 0.78 | .38 |
| *Note. df* = degrees of freedom, *M* = mean, *SD* = standard deviation. | | | | |

4. Supplementary Methods

*4.1 Stimuli*

During the fMRI experiment, the written sentences were presented on a screen, through a mirror mounted on the scanner head coil. The sentences were presented in the middle of the screen with white font (font: Arial, size: 14, viewing distance of ~40cm) on a grey background.

During the experiment the heard sentences were presented binaurally through insert earphones (Sensimetrics model S14; Sensimetrics, Malden, MA, USA, or KARADU audio stimulator, Unides Design Ay, depending on sample cohort). The intensity of the sound was set to a loud, but pleasant level for each participant individually (~80 dB SPL at the eardrum).

*4.2 fMRI procedure*

As each task block consisted of six congruent and six incongruent attended sentences, each of the eight different experimental conditions (2 tasks × 2 distractors × 2 congruence) were repeated six times in a block. Thus, the total number of trials in the whole experiment for each of the eight conditions was 18 (3 × 6).

The stimuli were randomized in the following way: The sentences were randomized into 3 sets (1 per run) that were identical for all participants. The order of sentences within a set was randomized for each participant. The order of the three sets was randomized and counterbalanced across participants.

During the measurements from 112 of the present participants in 2013–2015, five additional task blocks were used: a dual task, a visual task with music and an auditory task with pseudotext as distractors and visual and auditory localizers. However, these five conditions were not used in the current study. More details about the former measurements can be found in Moisala and colleagues (2016; 2018) reporting attention-related findings from the 2013–2015 measurements.

*4.3 fMRI preprocessing*

Preprocessing was carried out using fMRIprep pipelines resulting in co-registered preprocessed data on the fsaverage surface. The T1 image processing included brain tissue segmentation, spatial normalization, and brain surface reconstruction of the T1-weighted structural images. The functional BOLD images were motion corrected using MCFLIRT (Jenkinson et al. 2002), high pass filtered (128s cut-off) and slice timing corrected using 3dTshift from AFNI 20160207 (Cox and Hyde, 1997, RRID:SCR_005927). To generate confound time series, used to denoise the fMRI data (see below), the fMRI time series were resampled into standard MNI152NLin6Asym space (note: this data was only used to generate confound time series, used to denoise data). ICA-AROMA (Pruim et al. 2015) was performed on the preprocessed fMRI data in MNI space after removal of non-steady state volumes and spatial smoothing with an isotropic, Gaussian kernel of 6 mm^3^ FWHM (full-width half-maximum). Several confounding time series were calculated: framewise displacement (FD) and three region-wise global signals. FD is calculated for each functional run, using its implementation in Nipype (following the definitions by Power et al. 2014). The three global signals are extracted within the CSF, the WM, and the whole-brain masks. A reference volume was generated for each BOLD run and the BOLD reference was coregistered to the T1-weighted reference using bbregister (FreeSurfer, Greve and Fischl, 2009). The data was projected onto the FreeSurfer average surface, based on the participant’s own Freesurfer surface, using mri_vol2surf function and smoothed 5mm^2^. For connectivity analysis and Psychophysical interactions (PPI), ICA-AROMA detected noise components were ‘non-aggressively’ denoised from the preprocessed fMRI data (Pruim et al. 2015).

*4.4 fMRI analyses*

For first level analyses, a general linear model with regressors for each of the 8 experimental conditions (2 tasks × 2 distractors × 2 congruences), instructions, feedback and selected nuisance regressors derived from fMRIprep (6 motion parameters, regressors for white matter and cerebrospinal fluid signal, 4 CompCor regressors and cosine regressors) was fitted to the data. For the data measured 2013–2015 regressors for the additional experimental conditions were added.

Group-level whole-brain analyses were performed using Freesurfer’s mri_glmfit function using first level contrast images averaged across runs. Cluster forming threshold z was set at 4.5. Permutation was used to correct for multiple comparisons and p < .01 as permutated cluster significance, clusters smaller than 100 mm^2^ were discarded.

References

Cox RW, Hyde JS. Software tools for analysis and visualization of fMRI data. *NMR*

*Biomed.* 1997:10(4-5):171–178.

Greve DN, Fischl B. Accurate and robust brain image alignment using boundary-

based registration. *Neuroimage.* 2009:48(1):63–72.

Jenkinson M, Bannister P, Brady M, Smith S. Improved optimization for the robust and

accurate linear registration and motion correction of brain images. *Neuroimage.* 2002:17(2):825–841.

Moisala M, Salmela V, Carlson S, Salmela-Aro K, Lonka K, Hakkarainen K, Alho K. Neural activity patterns between different executive tasks are more similar in adulthood than in adolescence. *Brain Behav*. 2018:*8*(9).

Moisala M, Salmela V, Hietajärvi L, Salo E, Carlson S, Salonen O, Lonka K, Hakkarainen K, Salmela-Aro K, Alho K. Media multitasking is associated with distractibility and increased prefrontal activity in adolescents and young adults. *Neuroimage.* 2016:*134*:113–121.

Power JD, Mitra A, Laumann TO, Snyder AZ, Schlaggar BL, Petersen SE. Methods to detect, characterize, and remove motion artifact in resting state fMRI. *Neuroimage*. 2014:84:320–341.

Pruim RHR., Mennes M, van Rooij D, Llera A, Buitelaar JK, Christian F. ICA-AROMA: A robust ICA-based strategy for removing motion artifacts from fMRI data. *Neuroimage*. 2015:112:267–277.
